# Genetic diversity and population structure of Balkan goat populations in Serbia based on microsatellite and mtDNA markers

**DOI:** 10.5194/aab-69-143-2026

**Published:** 2026-03-09

**Authors:** Nina Dominiković, Minja Zorc, Marko Ristanić, Vladimir Dimitrijević, Jovan Blagojević, Uroš Glavinić, Zoran Stanimirović

**Affiliations:** 1 Department of Biology, Faculty of Veterinary Medicine, University of Belgrade, Bul. oslobodjenja 18,11000 Belgrade, Serbia; 2 Department of Animal Science, Biotechnical Faculty, University of Ljubljana, Jamnikarjeva 101, 1000 Ljubljana, Slovenia; 3 Department of Animal Breeding and Genetics, Faculty of Veterinary Medicine, University of Belgrade, Bul. oslobodjenja 18, 11000 Belgrade, Serbia

## Abstract

Indigenous breeds, such as the Balkan goat, represent valuable genetic resources due to their adaptation to specific ecological and socio-economic conditions. Goats are vital to rural livestock systems, particularly in regions with limited infrastructure, due to their resilience and low resource demands. This study presents a comprehensive genetic characterization of the Balkan goat in Serbia using microsatellite and mitochondrial DNA markers. Nuclear variation was assessed using 13 polymorphic microsatellite loci, revealing a mean expected heterozygosity of 0.756 and a mean polymorphic information content (PIC) of 0.721, indicating a high level of genetic diversity. The overall inbreeding coefficient (FIS =0.042 ) was low. Maternal lineage diversity was analyzed by sequencing the mitochondrial DNA control region in 42 individuals, identifying 23 distinct haplotypes predominantly belonging to haplogroup A, with a rare occurrence of haplogroup C. Together, the results demonstrate substantial genetic variation within the population at both nuclear and mitochondrial levels, providing essential insights for conservation and sustainable management of this autochthonous breed.

## 1 Introduction

Indigenous breeds play an important role in animal breeding due to their historical adaptation to specific ecological, sociocultural, and economic conditions (Tefiel et al., 2018). Goats (*Capra hircus*), as classified by the Food and Agriculture Organization (FAO), are among the five most significant domestic animal species, together with cattle, sheep, pigs, and horses (FAO, 2015). It was one of the first livestock species domesticated by humans, with domestication events traced back approximately 10 000 years in the Fertile Crescent. This process involved the transition from its wild ancestor, the bezoar goat (*Capra aegagrus*), originally inhabiting regions of present-day Iran and Anatolia. Early domestication efforts were driven by human selection for traits such as docility and resilience in harsh environments, which facilitated the global expansion and genetic diversification of the species (Colli et al., 2018). Compared to large ruminants, goats require fewer resources for feeding and housing, which enables their maintenance in environments with restricted economic capacity and limited agricultural infrastructure (Dixit et al., 2012; Lohani and Bhandari, 2021). They are, therefore, considered a vital resource for rural economies due to their capacity to adapt to difficult environmental conditions and to survive in areas with less resource availability.

Assessment of genetic diversity in local breeds is a fundamental step in the development of breeding programs and design of effective conservation strategies, as demonstrated in studies on cattle (Ristanić et al., 2020, 2024), sheep (Xia et al., 2021; Marković et al., 2022), pigs (Škorput et al., 2024), donkeys (Stanišić et al., 2017), dogs (Dimitrijević et al., 2020), and goats (Brito et al., 2017; Zhao et al., 2023). Effective management of animal genetic resources requires a comprehensive understanding of genetic variability, population structure, and spatial distribution of genetic markers (Asroush et al., 2018). Identifying intra- and inter-breed variation through genetic diversity characterization is of particular importance for maintaining the adaptability and productivity of domestic animal populations (El-Sayed et al., 2016; Hadi et al., 2020; Ristanić, 2022).

Molecular tools used in the assessment of genetic diversity include a range of markers, among which microsatellites, also known as short tandem repeats (STRs), have been extensively used over the last few decades due to their high polymorphism, widespread genomic distribution, co-dominant inheritance, and compatibility with automated genotyping techniques (Crispim et al., 2014; Laoun et al., 2020). In the context of conservation genetics of farm animals, including goats, microsatellites have been shown to be valuable markers enabling identification of inbreeding, population subdivision, and patterns of admixture (Marković et al., 2022; Whannou et al., 2023). Although single-nucleotide polymorphisms (SNPs) are becoming the markers of choice in genetic diversity studies, microsatellites still remain a recommended tool for an initial evaluation of the genetic structure in farm animals (Laoun et al., 2020). Mitochondrial DNA (mtDNA), particularly the hypervariable D-loop region, exhibits a high mutation rate compared to nuclear DNA, making it a marker relevant for studying maternal lineages, phylogenetic relationships, and historical patterns of domestication in goats (Guo et al., 2022). Analysis of mtDNA variation contributes to an understanding of domestication processes, population migrations, and identification of distinct genetic resources, which are important for the development of conservation and breeding strategies aimed at maintaining genetic diversity in goat populations (Masila et al., 2024). Phylogenetic studies of domestic goats have identified six well-supported maternal haplogroups distributed globally: A, B, and C (Luikart et al., 2001); D (Sultana et al., 2003); F (Sardina et al., 2006); and G (Naderi et al., 2007). The combined use of microsatellite markers and mtDNA sequences enables a comprehensive assessment of both biparental and maternal components of genetic variation, which is essential for the accurate characterization of goat populations. These molecular approaches provide complementary insights into population structure, levels of inbreeding, phylogenetic origin, and historical gene flow, all of which are fundamental for the conservation and sustainable management of caprine genetic resources (Selionova et al., 2021).

The Balkan goat and the Serbian white goat are two officially recognized autochthonous goat breeds in Serbia (Grittner et al., 2021). The Balkan goat is considered a native breed with notable phenotypic variability, including several ecotypes differentiated primarily by coat color. Previous studies on the breed have focused on phenotypic characteristics, including morphological and production traits. For example, Caro Petrović et al. (2012) analyzed the influence of genetic and environmental factors on growth traits in the Balkan goat without employing molecular tools, while Marković et al. (2020) evaluated milk production parameters in relation to environmental variables, again without incorporating DNA-based analyses. Though phenotypic diversity of this indigenous breed has been documented in previous studies, data on its genetic background are still lacking, and genetic analyses using molecular markers have not yet been conducted or reported. This study, therefore, aimed to obtain data on genetic variability, determine population structure, and assess maternal lineage diversity of the Balkan goat population. Combined mitochondrial DNA and microsatellite marker analyses were conducted to investigate both maternal lineage and nuclear genetic variation in the Balkan goat for the purposes of providing comprehensive data relevant for phylogenetic assessment, population structure analysis, and development of effective conservation strategies.

## 2 Materials and methods

### 2.1 Animals and sampling

**Figure 1:**
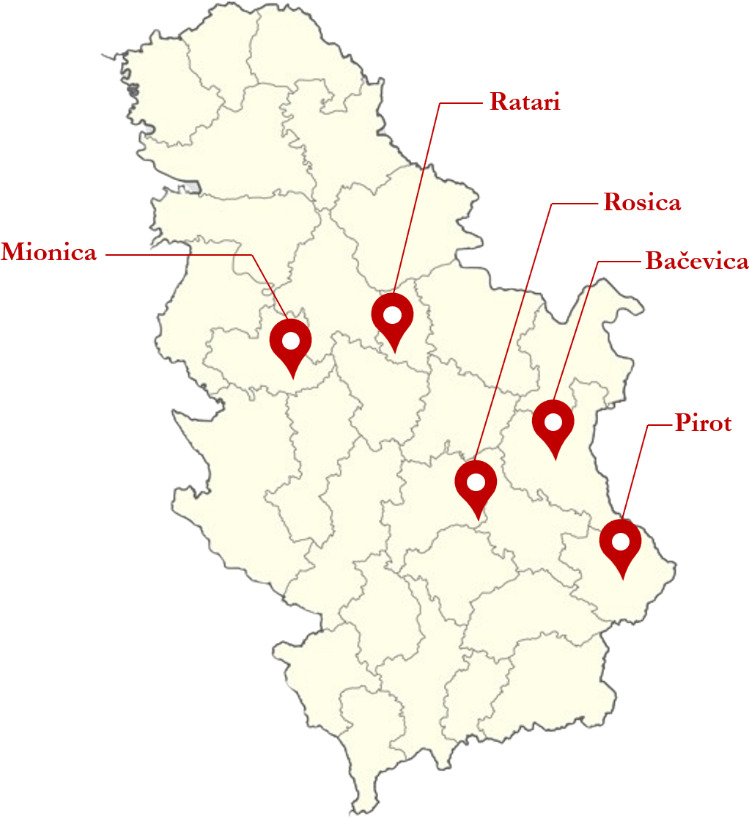
Geographical distribution of sampling sites for the Balkan goat population in Serbia. Sampling locations include Rosica (municipality of Crna Trava), Ratari (municipality of Smederevska Palanka), Bačevica (municipality of Boljevac), Mionica (western Serbia), and Pirot (southeastern Serbia).

The Balkan goat is an autochthonous goat breed of Serbia, mostly kept in central and southern Serbia. This was the only investigated goat breed in our study. To obtain a representative overview of the genetic diversity within the Balkan goat population, 72 biological samples were collected from multiple geographically distinct locations across Serbia. Blood samples were taken from adult individuals in the regions depicted in Fig. 1: 20 samples from Rosica (municipality of Crna Trava; 42°48^′^36.53^′′^ N, 22°17^′^56.43^′′^ E), 18 samples from Ratari (municipality of Smederevska Palanka; 44°21.98^′^ N, 20°57.39^′^E), 5 samples from Bačevica (municipality of Boljevac; 43°49^′^49.08^′′^ N, 21°57^′^11.16^′′^ E), 18 samples from Mionica (western Serbia; 44°15^′^ N, 20°05^′^ E), and 11 samples from Pirot (southeastern Serbia; 43°10^′^ N, 22°36^′^ E). The number of samples collected per location varied due to practical constraints; however, individuals were sampled randomly within each location. Larger flocks were therefore represented by a higher number of samples, whereas smaller flocks contributed fewer individuals. Additional variation in sample numbers among locations resulted from practical field constraints, including animal availability during sampling visits and differences in the amount and quality of collected biological material. In addition, animals representing different family lines were preferentially selected in order to minimize sampling of closely related individuals. To account for uneven sampling groups, clustering approaches such as STRUCTURE v2.3.4 (Pritchard et al., 2000) and the discriminant analysis of principal components (DAPC) were applied. Samples were collected from five flocks, each representing a single geographic location. Breed purity was confirmed through official breeder documentation and compliance with the recognized breed standard given by the FAO Domestic Animal Diversity Information System (DAD-IS) (FAO, 2010).

Blood samples were aseptically collected via jugular venipuncture and placed in 10 mL vacutainers containing an anticoagulant EDTA (BD Vacutainer, Plymouth, Devon, UK). The samples were then stored at 4 °C until DNA extraction. The DNA extraction of 72 whole-blood samples was performed using the commercial set MasterPure™ DNA Purification Kit for Blood Version II (Lucigen Corporation, USA) according to Ristanić et al. (2024). The extracted DNA was subsequently quantified using a UV–VIS spectrophotometer (BioSpec-nano, Shimadzu Scientific Instruments, Kyoto, Japan) to assess its quality and quantity. The extracted DNA samples were stored at -20 °C until further analysis.

### 2.2 Microsatellite genotyping

The microsatellite genotyping was done by multiplex amplification of sequences using the primers for 14 microsatellite markers recommended by the International Society for Animal Genetics/Food and Agriculture Organization (ISAG/FAO) for goat diversity analysis, including OarFCB20, MAF065, ILSTS008, INRA023, CSRD247, SRCRSP23, ILSTS87, INRA063, SRCRSP08, INRA005, McM527, INRA006, ILSTS19, and SRCRSP05 (Table 1). Forward primers were fluorescently labeled with 6-FAM, VIC, NED, or PET dyes (Table 1) to enable multiplex fragment analysis. Fragment separation was performed on a capillary sequencer ABI 3500 DNA Analyzer (Applied Biosystems, Foster City, CA, USA), and electropherograms were analyzed using GeneMapper software (Applied Biosystems, Foster City, CA, USA).

**Table 1:** Primer sequences of the 14 microsatellite loci used for genotyping.

Marker	Chromosome	Accession	Primer sequence ( $5^{\prime }\to 3^{\prime }$ )	Fluorescent	Reference
		number	forward, reverse R	dye	
CSRD247	OAR14	EU009450	GGACTTGCCAGAACTCTGCAAT	6-FAM	Kemp et al. (1995)
			CACTGTGGTTTGTATTAGTCAGG		
ILSTS008	OAR9	L23483	GAATCATGGATTTTCTGGGG	6-FAM	Kemp et al. (1993a)
			TAGCAGTGAGTGAGGTTGGC		
ILSTS19	BTA21	L23492	AGGGACCTCATGTAGAAGC	PET	Kemp et al. (1993b)
			ACTTTTGGACCCTGTAGTGC		
ILSTS87	BTA28	L37279	AGCAGACATGATGACTCAGC	VIC	Kemp et al. (1995)
			CTGCCTCTTTTCTTGAGAGC		
INRA005	BTA12	X63793	TTCAGGCATACCCTACACCACATG	NED	Vaiman et al. (1992)
			AAATATTAGCCAACTGAAAACTGGG		
INRA006	BTA3	X63793	AGGAATATCTGTATCAACCGCAGTC	PET	Vaiman et al. (1992)
			CTGAGCTGGGGTGGGAGCTATAAATA		
INRA023	BTA3	X67830	GAGTAGAGCTACAAGATAAACTTC	6-FAM	Vaiman et al. (1994a)
			TAACTACAGGGTGTTAGATGAACTC		
INRA063	BTA18	X71507	GACCACAAAGGGATTTGCACAAGC	VIC	Vaiman et al. (1994b)
			AAACCACAGAAATGCTTGGAAG		
MAF65	OAR15	M67437	AAAGGCCAGAGTATGCAATTAGGAG	6-FAM	Buchanan et al. (1994)
			CCACTCCTCCTGAGAATATAACATG		
MCM527	OAR5	EU009450	GTCCATTGCCTCAAATCAATTC	NED	Crawford et al. (1995)
			AAACCACTTGACTACTCCCCAA		
OARFCB20	OAR2	L20004	GGAAAACCCCCATATATACCTATAC	6-FAM	Buchanan et al. (1994)
			AAATGTGTTTAAGATTCCATACATGTG		
SRCRSP5	CHI21	L22197	GGACTCTACCAACTGAGCTACAAG	PET	Arevalo et al. (1994)
			TGAAATGAAGCTAAAGCAATGC		
SRCRSP8	CHI (unknown)	L22193	TGCGGTCTGGTTCTGATTTCAC	VIC	Bhebhe et al. (1994)
			CCTGCATGAGAAAGTCGATGCTTAG		
SRCRSP23	CHI (unknown)		TGAACGGGTAAAGATGTG	VIC	Yeh et al. (1997)
			TGTTTTTAATGGCTGAGTAG		

#### Microsatellite data analysis

To test for the presence of null alleles, large allele dropout, and scoring errors, we used Micro-Checker v2.2.3 (Van Oosterhout et al., 2004), applying 99 % confidence intervals for Monte Carlo simulations. Cervus v3.0.7 was used to calculate standard genetic diversity parameters, such as observed and expected heterozygosity, polymorphic information content (PIC), number of alleles per locus, and null allele frequencies. Inbreeding coefficients (FIS) were calculated per locus and across loci using FSTAT v2.9.4 (Goudet, 2003).

Effective population size (Ne) was estimated using NeEstimator v2.1, based on the linkage disequilibrium method (Do et al., 2014) with different minor allele frequency thresholds (0.05, 0.02, 0.01, and no threshold). Additionally, the heterozygote excess and molecular coancestry methods were applied for Ne estimation.

To assess recent reductions in effective population size, we performed a bottleneck analysis using BOTTLENECK v1.2.02 (Piry et al., 1999). Three statistical tests were applied: (1) the Wilcoxon signed-rank test, (2) the sign test, and (3) the standardized differences test, each run under both the infinite allele model (IAM) and the stepwise mutation model (SMM). We used 1000 replications for each test. Additionally, we examined the allele frequency distribution to identify mode-shift distortions indicative of recent bottlenecks. Due to small sample sizes, bottleneck tests were only performed on the pooled dataset, not on individual subpopulations.

To investigate population structure, we conducted Bayesian clustering analysis using STRUCTURE based on 13 microsatellite loci. We applied the admixture model with correlated allele frequencies. The number of assumed clusters ( K ) ranged from 1 to 8, with 10 independent runs per K . Each run was performed using a burn-in period of 100 000 iterations followed by 500 000 Markov chain Monte Carlo (MCMC) iterations. The optimal number of clusters was determined using StructureSelector (Li and Liu, 2018), which implements the Evanno ΔK statistic (Evanno et al., 2005), evaluates mean log-likelihood values [Ln P ( D )] (Pritchard et al., 2000), and applies the Puechmaille estimators (Puechmaille, 2016) for an inference of K .

We also performed DAPC in R using the adegenet package (Jombart, 2008) to explore genetic structure without assuming Hardy–Weinberg equilibrium. The number of clusters ( K ) was inferred using the “find.clusters” function based on the lowest Bayesian information criterion (BIC). The DAPC was then conducted with 20 principal components as determined by a-score optimization, and the results were visualized using scatterplots of the first two discriminant functions.

### 2.3 MtDNA amplification and sequencing

For mitochondrial DNA analysis, we selected 42 samples, ensuring representation of all sampling locations, as shown in a previous study of Drzaic et al. (2019). Thermal cycling conditions and primers for amplification of the 725 bp fragment of the mtDNA control region were adopted from Amills et al. (2004); the forward primer was 5'-CGC TCG CCT ACA CACAAA T-3', and the reverse primer was 5'-AAT GCC CAT GCC TAC CAT TA-3'. PCR products were separated by electrophoresis on a 1% agarose gel. Sequencing was performed on an ABI PRISM® 3100-Avant Genetic Analyzer (Applied Biosystems, USA) using the ABI Prism BigDye Terminator v3.1 Cycle Sequencing kit. The same primers were used for both PCR amplification and sequencing on both strands.

#### MtDNA data analysis

All the chromatograms were visualized and manually inspected using CLC Genomics workbench 25.0.1 software (Qiagen). Sequence alignments were generated using the ClustalW algorithm (Larkin et al., 2007). A phylogenetic tree was inferred and visualized in CLC Genomics Workbench based on the aligned sequences. Genetic diversity parameters, including the number of haplotypes, haplotype diversity, nucleotide diversity, and the number of polymorphic sites, were calculated using DnaSP v6 (Rozas et al., 2017). The mitochondrial D-loop sequences generated in this study have been deposited in GenBank under accession numbers PX713058–PX713080.

## 3 Results

### 3.1 Genetic diversity based on microsatellite markers

We genotyped 72 goats at 14 microsatellite loci recommended by the ISAG/FAO panel to assess genetic diversity in the Balkan goat population. Samples with incomplete genotype data were excluded, resulting in a final dataset of 69 individuals used for analysis. To ensure the reliability of downstream analyses, we first evaluated the presence of null alleles. Null allele frequencies were estimated for each microsatellite locus using the Oosterhout, Chakraborty, Brookfield 1, and Brookfield 2 methods (Table 2). The majority of loci displayed low or negative estimates across all methods. The highest estimate was observed at ILSTS19, with a Brookfield 2 value of 0.120, which may indicate the presence of a null allele at this locus. ILSTS19 was therefore excluded from subsequent analyses.

**Table 2:** Null allele frequency estimates for 14 microsatellite loci based on four different methods.

Locus	Null present	Oosterhout	Chakraborty	Brookfield 1	Brookfield 2
OarFCB20	no	-0.041	-0.034	-0.030	0.000
MAF065	no	-0.014	-0.011	-0.011	0.000
ILSTS008	no	-0.016	-0.009	-0.007	0.000
INRA023	no	-0.023	-0.017	-0.015	0.000
CSRD247	no	-0.012	-0.016	-0.014	0.000
SRCRSP23	no	0.000	0.001	0.000	0.000
ILSTS87	no	0.064	0.067	0.055	0.055
INRA063	no	0.054	0.061	0.047	0.047
SRCRSP08	no	-0.011	0.001	0.001	0.001
INRA005	no	0.012	0.009	0.007	0.007
McM527	no	-0.005	0.000	0.000	0.000
INRA006	no	0.035	0.038	0.034	0.034
ILSTS19	yes	0.093	0.106	0.086	0.120
SRCRSP05	no	0.061	0.059	0.048	0.048

After excluding ILSTS19, genetic diversity parameters were calculated across the remaining 13 microsatellite loci (Table 3). All retained loci were polymorphic, with the number of alleles per locus ranging from 5 (INRA005) to 14 (SRCRSP23) and a mean of 8.69 alleles per locus. The mean expected heterozygosity across loci was 0.756, and the mean polymorphic information content (PIC) was 0.721, indicating a high level of genetic variability within the population. Observed heterozygosity ranged from 0.594 (ILSTS008) to 0.870 (MAF065, SRCRSP23), while expected heterozygosity ranged from 0.588 to 0.877. Tests for Hardy–Weinberg equilibrium did not reveal significant deviations after Bonferroni correction.

**Table 3:** Genetic diversity parameters for 13 microsatellite loci. k – number of alleles per locus, N – number of genotyped individuals, $H_{O}$ – observed heterozygosity, $H_{E}$ – expected heterozygosity under Hardy–Weinberg equilibrium, PIC – polymorphic information content, HW – result of Hardy–Weinberg equilibrium test (NS – not significant, ND – not determined), FIS – inbreeding coefficient.

Locus	k	N	$H_{O}$	$H_{E}$	PIC	HW	FIS
OarFCB20	7	69	0.797	0.750	0.702	NS	-0.064
MAF065	11	69	0.870	0.856	0.833	ND	-0.016
ILSTS008	7	69	0.594	0.588	0.561	NS	-0.011
INRA023	10	69	0.754	0.733	0.698	NS	-0.028
CSRD247	9	69	0.783	0.763	0.727	NS	-0.026
SRCRSP23	14	69	0.870	0.877	0.857	ND	0.008
ILSTS87	8	69	0.696	0.801	0.766	NS	0.132
INRA063	7	69	0.609	0.693	0.636	NS	0.123
SRCRSP08	10	69	0.710	0.717	0.688	NS	0.009
INRA005	5	69	0.638	0.654	0.594	NS	0.026
McM527	8	69	0.754	0.758	0.720	NS	0.006
INRA006	9	69	0.797	0.867	0.845	ND	0.081
SRCRSP05	8	69	0.681	0.772	0.739	NS	0.118

Inbreeding coefficients (FIS) calculated per locus revealed mostly low or negative values. The overall multilocus FIS value was 0.042, indicating a slight but not substantial deficit of heterozygotes, which may reflect subtle inbreeding or population substructure. The highest positive FIS values were observed at ILSTS87 (0.132), INRA063 (0.123), and SRCRSP05 (0.118).

### 3.2 Effective population size (Ne) estimation

The estimated Ne at a minor allele frequency threshold of 0.05 was 36.2 (95 % CI: 30.5–43.4). Lowering the allele frequency threshold slightly increased Ne estimates, reaching 45.9 (39.6–53.8) at 0.02, 47.3 (41.1–54.9) at 0.01, and 59.5 (51.0–70.5) with no filtering. Both the heterozygote excess and the molecular coancestry methods yielded infinite Ne values, indicating an absence of recent bottlenecks or high inbreeding within the sample. 

### 3.3 Bottleneck analysis

Evidence of a possible historical bottleneck was detected in the Balkan goat population. A significant heterozygosity excess was observed under the infinite allele model (IAM; Wilcoxon test p=0.016 ), suggesting a past reduction in effective population size. However, no signal of a bottleneck was detected under the stepwise mutation model (SMM), and the allele frequency distribution retained a normal L-shaped pattern, which indicates the absence of a strong recent bottleneck. Due to the small sample sizes within individual subpopulations, bottleneck analyses were not separately performed for each group.

**Figure 2:**
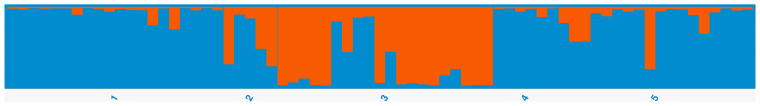
STRUCTURE bar plot at K=2 showing the assignment of individuals from five sampling locations (1 – Rosica, 2 – Bačevica, 3 – Ratari, 4 – Pirot, 5 – Mionica) into two main genetic clusters. Each vertical bar represents an individual, and colors indicate cluster membership proportions.

### 3.4 Population structure

STRUCTURE analysis (Fig. 2) suggested a weak signal of population structure. Evaluation using the Evanno ΔK method indicated K=2 as the uppermost hierarchical level, with a maximum ΔK value of 53.46 and a high mean similarity score among replicates (0.987). This ΔK peak resulted from a sharp increase in mean log-likelihood between K=1 (LnP =-3082.49±1.17 ) and K=2 (LnP =-2974.75±1.49 ). Inspection of mean log-likelihood values [Ln P ( D )] showed that ln-likelihoods continued to increase only gradually for higher K , reaching -2946.66±12.62 at K=3 , -2926.52±52.26 at K=4 , and -2907.33±60.50 at K=5 . The sharp rise in standard deviations for K≥4 indicated poorly supported clustering. In contrast to the ΔK result, all four Puechmaille estimators, MedMedK =1 , MedMeanK =1 , MaxMedK =1 , and MaxMeanK =1 , identified K=1 as the optimal number of clusters, indicating the strongest support for no population subdivision.

**Figure 3:**
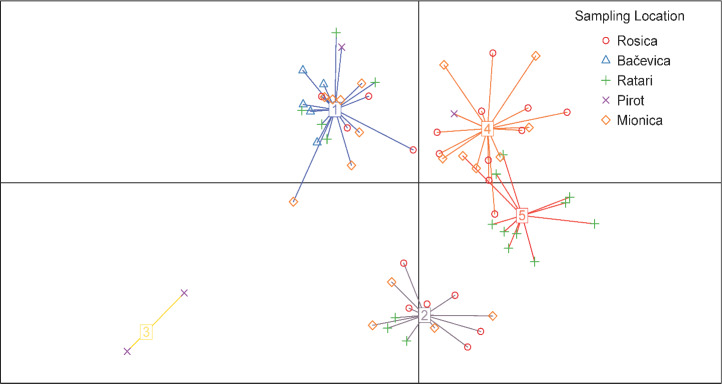
Discriminant analysis of principal component (DAPC) scatterplot showing five genetic clusters among 69 Balkan goats. Each point represents an individual, colored and shaped according to sampling location (Rosica, Bačevica, Ratari, Pirot, Mionica), while cluster assignment (numbers 1–5) is based on genetic similarity. The analysis reveals partial overlap between sampling locations and genetic clusters.

Discriminant analysis of principal components (DAPC) identified five genetic clusters based on the lowest Bayesian information criterion (BIC) value. Five clusters were generally well separated, with partial overlap observed only between clusters 4 and 5, indicating moderate population structure within the dataset (Fig. 3).

Overall, both analyses indicate weak-to-moderate genetic structuring: STRUCTURE supports at most a shallow split ( K=2 ), while DAPC suggests finer-scale differentiation among sampling locations, consistent with partial isolation and uneven sampling across flocks.

**Figure 4:**
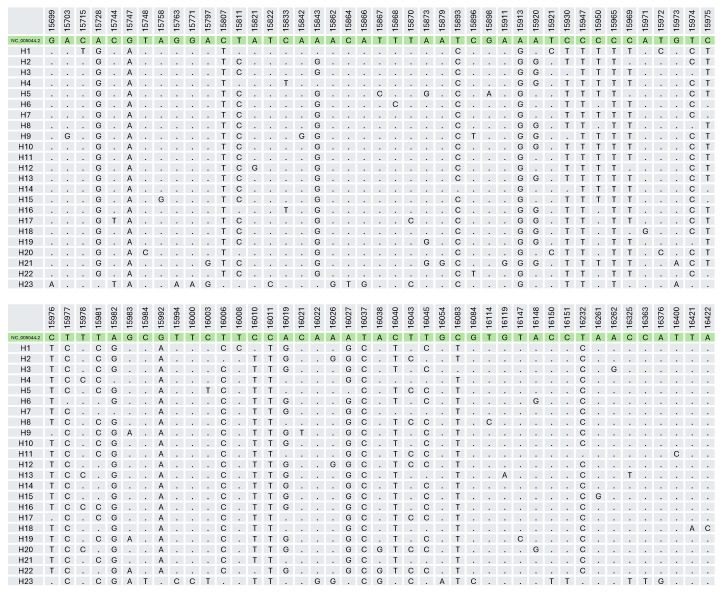
Distribution of 80 polymorphic sites (725 bp sequences) found in 23 Balkan goat haplotypes. The reference sequence GenBank accession is NC_005044.2.

### 3.5 Mitochondrial DNA diversity

In the sample of 42 Balkan goats, sequencing of a 725 bp mitochondrial DNA region revealed 80 polymorphic sites, including 32 singleton variable sites and 48 parsimony informative sites. Mitochondrial D-loop sequence analysis identified 23 distinct haplotypes among the analyzed goat samples (Table 4). Nine haplotypes were shared by multiple individuals: H6 was the most frequent ( n=7 ), followed by H5 ( n=4 ); H8, H9, and H12 ( n=3 each); and H3, H7, H11, and H13 ( n=2 each). The remaining 14 haplotypes were unique. Mitochondrial D-loop haplotypes showed high similarity to previously published *Capra hircus* sequences in GenBank. The closest matches differed by 1–5 nucleotide substitutions across the full aligned fragment (725 bp). The haplotype H10 (PX713059) showed the highest similarity, differing by a single-nucleotide substitution from several GenBank sequences, including DQ121602 (Qianbeima, China), DQ121618 (Shaannan White, China), and LR884210 (goat from Aceramic Neolithic Ganj Dareh, Zagros Mountains, western Iran). H14 (PX713061) and H15 (PX713062) differed by two substitutions from sequences LS992600 (goat from early Neolithic site of Blagotin-Poljna, Serbia), MN786578 (Sarda, Italy), and KR059190 (Payoya, Spain). H3 (PX713058) differed by three substitutions from sequence MH621421 (Cameroon), whereas H13 (PX713060) differed by five substitutions from MN786588 (Sarda, Italy).

The analyzed control region exhibited high genetic variability, with a haplotype diversity ( $H_{d}$ ) of 0.954 (standard deviation (SD) =0.036 ) and a nucleotide diversity ( π ) of 0.017.

**Figure 5:**
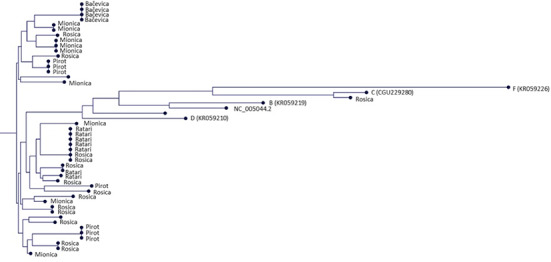
Phylogenetic tree based on mitochondrial DNA (D-loop) sequences of 42 Balkan goats sampled from different localities. Most individuals cluster within haplogroup A, while one individual from the Rosica locality is grouped within haplogroup C. Reference sequences representing major domestic goat haplogroups (B, C, D, and F) are included for comparison. The tree indicates high haplotypic diversity and weak geographic structuring, with individuals from multiple localities interspersed across branches.

The phylogenetic tree (Fig. 5) revealed 23 distinct mtDNA haplotypes among the 42 sampled Balkan goats, with no single dominant haplotype. Several haplotypes were shared between multiple geographic locations, such as Rosica, Ratari, and Pirot, while others appeared to be limited to specific regions (e.g., Bačevica and Mionica).

## 4 Discussion

The Balkan goat is Serbia's main autochthonous goat breed, yet its genetic diversity has not been previously explored. This study provides the first assessment of its genetic diversity and population structure using microsatellite and mtDNA markers. The results reveal high levels of nuclear and mitochondrial genetic variation, highlighting the breed's importance as a reservoir of locally adapted genetic resources.

Microsatellite analysis demonstrated substantial genetic variability, with an average of 8.96 alleles per locus and high mean expected heterozygosity ( $H_{E}=0.75$ 6) comparable to other European indigenous breeds, such as the Carpathian goat ( k=9.143 , $H_{E}=0.701$ , PIC =0.664 ) (Kawęcka et al., 2022). The average PIC of 0.721 reflects the informativeness of the marker panel, confirming its suitability for genetic monitoring of this breed. A similar level was reported in a study of an Italian goat population, which showed a mean PIC of 0.60 (Ceccobelli et al., 2020). The overall FIS value observed in the Balkan goat population (0.042) aligns with data from regional breeds such as Bulgarian goat populations, where low inbreeding coefficients (FIS =0.025 on average) were also recorded, suggesting minimal inbreeding and the preservation of genetic diversity in regional breeds (Yordanov et al., 2024), supporting the presence of substantial genetic diversity despite potential substructuring within local populations. These results collectively emphasize the preservation of genetic variability and the maintenance of healthy breeding structures in regional goat populations.

The estimated effective population size ( $N_{e}$ ) ranged from 36.2 at an MAF threshold of 0.05 to 59.5 with no allele frequency filtering, with intermediate estimates of 45.9 and 47.3 at MAF thresholds of 0.02 and 0.01, respectively. Thus, Ne increased as the MAF threshold was relaxed and more rare alleles were retained, illustrating the sensitivity of LD-based Ne estimators to the treatment of low-frequency alleles. Because all estimates were of the same order of magnitude and showed overlapping confidence intervals, we interpret these results as indicating a moderately low Ne of approximately 40–50 breeding individuals. These moderate values are typical for small, regionally distributed populations. Such populations are at risk of losing rare alleles through drift, emphasizing the need for careful conservation planning to preserve genetic variation and avoid future decline (Michailidou et al., 2019). Bottleneck analysis indicated possible signatures of a historical demographic contraction, with significant heterozygosity excess under the infinite allele model (Demir, 2024). However, the lack of significance under the stepwise mutation model and the maintenance of a normal L-shaped allele frequency distribution suggest that no strong recent bottleneck has occurred. This combination of results is indicative of a past reduction in population size, followed by demographic recovery or continued gene flow among subpopulations (Demir, 2024). Bayesian clustering (STRUCTURE) and multivariate analysis (DAPC) both identified population substructure. STRUCTURE supported two major genetic clusters ( K=2 ). However, different clustering criteria indicated that the detected subdivision was weak. While the Evanno ΔK method identified K=2 , all four Puechmaille estimators supported K=1 , suggesting that the population is largely genetically homogeneous and that the ΔK peak reflects only a shallow hierarchical split rather than clearly separated subpopulations. In contrast, DAPC partitioned the dataset into five clusters. These clusters most likely represent local differentiation among flocks or geographic regions. The combined results indicate weak-to-moderate population structuring accompanied by ongoing gene flow, which is consistent with breeding practices and animal exchange among farms. The observed substructure, along with low FIS values, implies the presence of limited gene flow and/or microevolutionary processes acting at the regional level. These patterns should be accounted for in breeding and conservation programs to maintain both overall diversity and local adaptations.

Mitochondrial DNA analysis revealed considerable maternal diversity predominantly within haplogroup A, with a rare haplogroup C lineage, with 23 haplotypes identified among only 42 individuals (Hd =0.953 ). Globally, domestic goats are partitioned into six major mtDNA haplogroups (A, B, C, D, F, and G), which differ in their geographic frequencies (Naderi et al., 2007). Haplogroup A is by far the most widespread lineage and predominates across Europe, the Middle East, Africa, and large parts of Asia. Haplogroup B is reported more frequently in east and southeast Asia, whereas haplogroup C occurs at low frequencies and has been detected sporadically in European and Asian populations. Haplogroups D and G are generally rare and are more commonly associated with regions closer to the domestication center in southwest Asia, while haplogroup F is typically infrequent and has been described in a limited number of populations (Luikart et al., 2001). In our dataset, the strong predominance of haplogroup A and the detection of a single haplogroup C lineage are consistent with this broadly uneven global distribution. The strongly uneven frequencies of haplogroups and the global predominance of haplogroup A reflect early domestication history followed by widespread human-mediated movements and admixture (Peng et al., 2022). The presence of multiple unique and shared haplotypes across regions and the star-like pattern in the haplotype network suggest historical population expansion from a common maternal ancestor and retention of ancient variation (Masuko et al., 2025). Our findings are consistent with studies on neighboring and distant populations. For example, high haplotype diversity within haplogroup A was similarly found in the Croatian spotted goat (Hd =0.967 ; Drzaic et al., 2019), Sardinian goats (Dettori et al., 2020), and several Albanian local breeds, where haplotype diversity ranged from 0.864 to 1.000 with an overall Hd =0.996 (Hoda et al., 2014). The predominance of haplogroup A, together with the detection of a rare haplogroup C lineage, may reflect a narrow maternal origin, founder effects, or long-term reproductive isolation. However, the high haplotype diversity indicates that significant variation has been maintained within this lineage. Similar patterns have been observed in goat populations worldwide, where haplogroup A dominates but harbors a deep evolutionary structure (Yi et al., 2022). This consistency supports the hypothesis of a widespread expansion from early domestication centers in the Fertile Crescent, followed by localized differentiation.

Preserving haplotype diversity within major maternal lineages is an important aspect of conservation genetics, even when only a single haplogroup is present (De et al., 2023). The predominance of haplogroup A and the high haplotype diversity observed in the Balkan goat population are consistent with global findings. A large-scale study of 4189 mitochondrial D-loop sequences showed that haplogroup A is the most common maternal lineage worldwide, including in European goat populations, where it accounts for approximately 89.7 % of samples (Peng et al., 2022). These results suggest that high levels of maternal genetic diversity can exist even within a single dominant haplogroup. Interestingly, the occurrence of comparable maternal signatures in both nearby and geographically distant goat breeds suggests a shared evolutionary background rooted in early domestication. This pattern likely stems from the initial expansion of female lineages from the Fertile Crescent, followed by regional differentiation without substantial introgression from divergent sources. In contrast, the phylogenetic analysis of Arabian goats revealed three distinct haplogroups (A, F, and G) (Naderi et al., 2007), indicating a more diverse maternal ancestry. The low frequency of haplogroups other than A (i.e., a single C lineage) in the Balkan goat population may reflect a narrow maternal origin, a historical bottleneck, or long-term reproductive isolation. This limited haplogroup diversity also suggests limited introgression from genetically divergent populations. These results reinforce the utility of mtDNA in tracing lineage divergence and identifying unique genetic resources. From a conservation perspective, the predominance of a single maternal lineage underscores the importance of preserving intra-lineage genetic diversity through carefully designed breeding programs, particularly in small, autochthonous populations that may be vulnerable to further loss of genetic variability.

## 5 Conclusions

The Balkan goat population displays substantial nuclear and mitochondrial genetic diversity, moderate effective population size, historical demographic fluctuations, and evident population substructure. These findings emphasize the need for structured, regionally sensitive conservation strategies that preserve both overall variability and locally adapted subpopulations. Integrating these molecular data into breeding programs will support the sustainable use and preservation of this important indigenous genetic resource. The results obtained will serve as a basis for the conservation of this autochthonous genetic resource in the Balkan region and for the development of scientifically informed breeding strategies aimed at improving and sustainably managing the Balkan goat breed.

## Data Availability

The data used and analyzed during this study are available from the corresponding author upon request.
